# Bariatric Surgery Is Associated With Reduced Incidence of Mild Cognitive Impairment and Alzheimer Disease and Related Dementias: A Retrospective Cohort Study

**DOI:** 10.1097/AS9.0000000000000541

**Published:** 2025-01-10

**Authors:** You Chen, Yubo Feng, Xinmeng Zhang, Katherine A. Gifford, Yasser Elmanzalawi, Jason Samuels, Vance L. Albaugh, Wayne J. English, Charles R. Flynn, Danxia Yu, Rui Zhang, Sayeed Ikramuddin

**Affiliations:** From the * Department of Biomedical Informatics, Vanderbilt University Medical Center, Nashville, TN; † Department of Computer Science, Vanderbilt University, Nashville, TN; ‡ Department of Neurology, Vanderbilt University Medical Center, Nashville, TN; § Department of Population Health Sciences, Geisinger, Danville, PA; ‖ Department of Surgery, Vanderbilt University School of Medicine, Nashville, TN; ¶ Metamor Institute, Pennington Biomedical Research Center, Baton Rouge, LA; # Division of Epidemiology, Department of Medicine, Vanderbilt University Medical Center, Nashville, TN; ** Department of Surgery, University of Minnesota, Minneapolis, MN.

**Keywords:** Alzheimer disease and related dementias, bariatric surgery, electronic health records, mild cognitive impairment

## Abstract

**Objective:**

To evaluate the association of bariatric surgery with reduced incidence and delayed development of mild cognitive impairment (MCI) and Alzheimer disease and related dementias (ADRD) in patients with obesity.

**Background:**

This retrospective longitudinal study utilized Electronic Health Records from Vanderbilt University Medical Center, covering 5303 patients who underwent bariatric surgery and 10,606 propensity score-matched obese patients who did not, from 2000 to 2023. Patients with prior MCI, ADRD, schizophrenia, alcoholism, gastric cancer, gastric ulcers, inflammatory bowel disease, coagulopathy, stroke, Parkinson disease, or brain cancer were excluded from both groups.

**Methods:**

Differences in time to MCI/ADRD between surgical and control groups were analyzed using linear regression, and adjusted for confounders: demographics, medical history, and socioeconomic status. Survival probability differences for MCI and ADRD between the 2 groups over time were assessed using Kaplan-Meier curves and log-rank tests. Incidence differences of MCI and ADRD between the groups were evaluated using Fine-Gray subdistribution hazard models, accounting for the competing risk of death and confounders.

**Results:**

Bariatric surgery was associated with a significantly reduced incidence of ADRD, evidenced by a subdistribution hazard ratio (SHR) of 0.37 (95% confidence interval [CI]: 0.15–0.89; *P* = 0.03). Similarly, the incidence of MCI was significantly lower in the surgical group, with an SHR of 0.57 (95% CI: 0.39–0.85; *P* = 0.01). Additionally, patients who underwent bariatric surgery experienced a delay of 2.01 years before developing MCI compared with the control group (95% CI: 0.70–3.50; *P* = 0.004).

**Conclusions:**

These findings suggest that bariatric surgery may serve as an effective strategy to delay the onset of MCI and reduce the risk of both MCI and ADRD in patients with obesity.

## INTRODUCTION

Obesity remains a critical public health challenge in the United States, with 42% of adults considered to have obesity (body mass index [BMI] ≥ 30 kg/m^2^) and 9.2% to have severe obesity (BMI ≥ 40 kg/m^2^).^1^ Obesity is increasingly acknowledged as a significant, independent risk factor for dementia, with numerous studies highlighting its impact on cognitive health.^2–5^ Notably, a comprehensive meta-analysis encompassing 19 studies with 589,649 participants and 2040 incident dementia cases with follow-up in subjects for up to 42 years delineated that having midlife obesity (BMI ≥ 30 kg/m^2^) substantially increases the risk of developing dementia in later life (relative risk [RR], 1.33; 95% confidence interval [CI], 1.08–1.63), compared with overweight status (25 kg/m^2^ < BMI < 30 kg/m^2^), which showed no significant association (RR, 1.07; 95% CI, 0.96–1.20).^2^ Evidence from the Framingham study showed the linkage between having obesity and Alzheimer’s disease (AD)-related gene expression, even when controlling for cardiovascular disease risk factors.^5^ Moreover, a projection model based on an Australian cohort indicated that reducing the prevalence of having midlife obesity to 20% and increasing the proportion of those individuals with a normal weight to 40% between 2015 and 2025 could result in a 10% decrease in dementia cases among individuals aged 65 to 69 years by 2050, compared with no intervention to stop current obesity trends.^6^

The most effective and durable therapy to manage severe obesity is bariatric surgery. Bariatric surgery achieves approximately 30% of total body weight loss and mediates medium to long-term control of comorbid illnesses, increasing interest regarding its potential impact on Alzheimer disease and related dementias (ADRD).^7–10^ It would also be important to understand if significant weight loss through current surgical procedures (ie, sleeve gastrectomy [SG] or Roux-en-Y gastric bypass [RYGB]) can mitigate the risk of future mild cognitive impairment (MCI) and progression of MCI to dementia.

Recent research offers mixed findings on the cognitive outcomes following bariatric surgery. For instance, patients with obesity experienced a significant increase in cognitive function postsurgery, with a 17% improvement observed 2 weeks after the procedure and a 21% improvement after 14 weeks, compared with normal-weight controls.^11^ Patients, 6 months following SG, were found to have cognitive enhancement compared to the baseline.^12^ Conversely, other studies have reported associations between bariatric surgery and an increased incidence of ADRD. In a cohort study of 51,078 subjects (matched in a 1:2 ratio for case to control) with an average participant age of 42 years at surgery and a predominance of female participants (78%), a higher incidence of dementia was reported in the surgery group (1.4%) compared with the control group (0.5%) over an average follow-up period of 10.5 years.^13^ Cox regression analysis, adjusted for a range of covariates such as sex, race/ethnicity, age at surgery or matched baseline year, and BMI category at surgery or matched baseline year, revealed a significantly increased risk of dementia in the surgery group (hazard ratio [HR] = 1.33, *P* = 0.02).^13^ Importantly, this study did not exclude patients with histories of conditions such as schizophrenia, alcoholism, gastric cancer, gastric ulcers, inflammatory bowel disease, and coagulopathy at baseline. These conditions are usually seen as reasons to avoid bariatric surgery or might indicate the need for surgery for reasons like gastrointestinal reconstruction rather than for weight or metabolic control.^14^ Furthermore, the study did not remove cases of stroke, Parkinson disease, or brain cancer, all of which are recognized potential confounders that could influence the evaluation of bariatric surgery’s effect on cognitive outcomes.^15^

The divergent outcomes highlight the complexities involved in understanding the relationship between bariatric surgery and subsequent cognitive impairment, emphasizing the critical need for comprehensive, longitudinal studies. Access to electronic health records (EHRs) with follow-ups extending up to 23 years at a single institution presents a unique opportunity to examine the medium to long-term impact of bariatric surgery on the incidence of ADRD, MCI, and transition from MCI to ADRD. This retrospective longitudinal cohort study allows for a detailed analysis over extended periods, providing insights into the temporal progression of cognitive changes and the potential long-lasting effects of bariatric surgery on cognitive health.

## MATERIALS AND METHODS

This study was approved by the Institutional Review Boards (IRBs) at Vanderbilt University Medical Center (VUMC) under IRB#221459. A full waiver of written informed consent from patients was granted by the IRB due to the fact that this study is retrospective with minimal risks to patients.

### Study Settings

We conducted a retrospective longitudinal cohort study at VUMC, a large academic medical center in Nashville, TN, USA, which provides primary and specialty referral care to patients from across the Southeast. The VUMC Surgical Weight Loss Program holds accreditation from the Metabolic and Bariatric Surgery Accreditation and Quality Improvement Program.^16–18^ Patients who underwent bariatric surgery were identified using the VUMC Bariatric Surgery Quality, Efficacy, and Safety registry data.

### Data and Study Cohorts

This study analyzed patients who underwent their first bariatric surgery at VUMC between December 2000 and May 2023. We focused solely on the initial bariatric procedure for each patient, excluding any later surgical revisions due to their complex nature. The date of the first bariatric surgery for each patient was designated as the index date, and the surgery itself was identified as the index visit. Patients who underwent bariatric surgery were identified using the Quality, Efficacy, and Safety registry data to form the surgery group.

Exclusion criteria were applied to both surgery and following matched control groups for patients with prior diagnoses of schizophrenia, alcoholism, gastric cancer, gastric ulcers, inflammatory bowel disease, or coagulopathy. These diagnoses were considered an exclusion for bariatric surgery or maybe the primary indicator for surgery for gastrointestinal reconstruction.

Additionally, individuals diagnosed with MCI and ADRD (see International Classification of Diseases (ICD) codes in Outcome Definition) before the baseline visits were also excluded. This approach was taken because the objective of the project is to examine the medium to long-term effects of bariatric surgery on the development of MCI and ADRD.

Furthermore, patients with a history of stroke (ICD-9/10-Clinical Modification (ICD-9/10-CM) codes: 431, 432, 433.x1, 434.x1, I61, I62, I63), Parkinson disease (ICD-9/10-CM codes: 332.0, G20), and brain cancer (ICD-9/10-CM codes: 191.9, C71.9) before the baseline visits were excluded due to the known association of these conditions with dementia. This careful selection process ensures the study’s focus remains on evaluating the specific effect of bariatric surgery on cognitive outcomes.

### Outcome Definition

There is no universally accepted standard for diagnosing ADRD and MCI. In this study, we employed the most utilized criteria for identifying diagnoses of ADRD and MCI in EHRs.^19^ A case of ADRD was confirmed if a patient’s EHRs contained at least 2 postbaseline visits, with each visit recording any of the following ICD-9/10 codes: Alzheimer disease (331.0, G30.0, G30.1, G30.8, G30.9), vascular dementia (290.40, 290.41, F01.50, F01.51), Lewy body dementia (331.82, G31.83), frontotemporal dementia (331.19, G31.09), and primary progressive aphasia (331.11, G31.01). Similarly, an MCI diagnosis was confirmed through an analogous method using the code set: 331.83, 294.9, G31.84, F09.

### Statistical Analysis

The longitudinal cohort study encompassed 5303 patients who underwent RYGB or SG (surgery group) and 10,606 propensity-matched obese patients who did not undergo the procedure (control group), covering the period from December 2000 to May 2023. Propensity score matching (PSM) was specifically applied to ensure comparable baseline characteristics between the surgery and control groups.^20^

The PSM score was generated using logistic regression, incorporating several key variables: the year of the baseline visit (±1 year), patient age at the year of the baseline visit (±1 year), exact BMI category before the baseline visit (across 4 ranges: 35–39.9, 40–44.9, 45–49.9, and ≥50 kg/m^2^), sex (female or male), race/ethnicity (Black, White, or others), the Charlson Comorbidity Index (CCI) before the baseline visit,^21^ the Area Deprivation Index (ADI) based on patient’s residence before the baseline visit (a larger ADI indicates a more deprived neighborhood),^22^ and the presence of type 2 diabetes (T2DM) before the baseline visit.^23,24^ PSM scores were then computed for individuals in both the surgery and control groups based on each variable to ensure a balanced comparison. A 1:2 case-to-control matching ratio was used to form the control cohort.

We employed Kaplan-Meier survival analysis, which shows the estimated percent survival of the subjects at each point in time,^25^ to track the development of differences in incidences of ADRD and MCI between the surgery and control groups over the follow-up periods. To assess the significance of these differences, log-rank tests, which test the null hypothesis that there is no difference between the 2 groups in the probability of an MCI/ADRD event at any time point,^26^ were utilized. Additionally, we employed Fine-Gray subdistribution hazard models to assess the association between bariatric surgery and the incidence of MCI/ADRD, adjusting for MCI/ADRD-related comorbidities that occurred within 1 year before the baseline visit and those that arose after baseline but before the MCI/ADRD incidence or before death/censoring. Baseline variables including age (in years), BMI category, sex, race/ethnicity, CCI, ADI, and presence of T2DM before baseline were also included in the analysis. We considered comorbidities occurring within 1 year before the baseline visit, including hypertension (ICD-9/10: 401.xx, I10),^27,28^ dyslipidemia (272.0, E78.0, E78.1, E78.2, E78.3, E78.4, E78.5),^29^ ischemic heart disease (IHD) (410–414, 429.2, I20–I25),^30,31^ major depressive disorders (296.2, 296.3, F32, F33),^32^ obstructive sleep apnea (OSA) (G47.33, 327.23),^33,34^ traumatic brain injury (TBI) (800.0–801.9, 803.0–804.9, 850.0–854.1, 959.01, 995.55, S02.0, S02.1, S02.8, S02.91, S04.02, S04.03, S04.04, S06, S07.1, T74.4),^35,36^ and glucagon-like peptide-1 (GLP-1) use based on the Observational Health Data Sciences and Informatics cohort 1038 (RxNorm IDs: 1551291, 1534763, 475968, 1991302, 60548, 1440051).^37^ We also included all the aforementioned comorbidities occurring after baseline but before the incidence of MCI/ADRD or death/censoring. Linear regression models were used to measure differences in time to MCI/ADRD between the surgical and control groups, excluding patients without follow-up MCI/ADRD incidences in both groups and adjusting for the same confounders as the Fine-Gray models. We measured weight loss at 3, 6, and 12 months; at 3, 5, and 10 years; and up to the time of ADRD/MCI diagnosis in both the surgery and control groups. Differences between groups were compared using the Mann-Whitney *U* test. To further analyze the association between the degree of weight loss and the incidence of ADRD/MCI in the surgery group, we conducted logistic regression models with the maximum total body weight loss within 2 years after surgery as the primary independent variable, adjusting for other baseline confounders. Additionally, to determine whether there are variations in ADRD/MCI risk between RYGB and SG surgeries, we performed chi-squared tests within the surgery group.

HRs for log-rank tests and logistic regression models and subdistribution hazard ratios (SHRs) for Fine-Gray models, along with their corresponding 95% CIs and *P* values, were reported. Statistical analyses were performed using R software (version 4.3.1), with a *P* value of less than 0.05 deemed to indicate statistical significance.

## RESULTS

Table 1 details participant summary statistics. Using PSM, we matched the bariatric surgery patients—3599 who underwent RYGB and 1704 who underwent SG—with control groups totaling 10,606 individuals, ensuring balance across key variables: BMI in 4 categories (35–39.9, 40–44.9, 45–49.9, and ≥50 kg/m^2^) (*P* > 0.99), age within 3 categories (18–45, 46–64, and ≥65) (*P* = 0.78), race (White, Black, and others) (*P* > 0.99), sex (*P* > 0.99), and T2DM (*P* > 0.99). Furthermore, the absolute standardized mean difference of 0.089 falls below the threshold of 0.1, indicative of a well-balanced PSM.^38^

**TABLE 1.** Characteristics of Bariatric Surgery and Matched Control Group

	Surgery (n = 5303)	Control (n = 10,606)	*P*
	No. Patient (%)		
Surgery			
RYGB	3599	NA	NA
SG	1704	NA	NA
Sex			
Female	4268 (80.5%)	8536 (80.5%)	>0.999*
Male	1035 (19.5%)	2070 (19.5%)	>0.999*
Race			
White	4344 (81.9%)	8688 (81.9%)	>0.999*
Black	922 (17.4%)	1844 (17.4%)	>0.999*
Others†	37 (0.7%)	74 (0.7%)	>0.999*
Baseline BMI			
BMI (35–40)	717 (13.5%)	1434 (13.5%)	>0.999*
BMI (40–45)	1598 (30.1%)	3196 (30.1%)	>0.999*
BMI (45–50)	1292 (24.4%)	2584 (24.4%)	>0.999*
BMI (>50)	1696 (32.0%)	3392 (32.0%)	>0.999*
T2DM baseline			
Yes	1074 (20.3%)	2148 (20.3%)	>0.999*
No	4229 (79.7%)	8458 (79.7%)	>0.999*
Age category baseline			
18–45	2770 (52.2%)	5538 (52.2%)	0.982*
46–64	2331 (44.0%)	4687 (44.2%)	0.778*
≥65	202 (3.8%)	381 (3.6%)	0.493*
Comorbidity within 1 y before the baseline			
Hypertension	3531 (66.6%)	4081 (38.5%)	**<0.001** *
Dyslipidemia	521 (9.8%)	307 (2.9%)	**<0.001** *
IHD	269 (5.1%)	544 (5.1%)	0.879*
Depression	1130 (21.3%)	810 (7.6%)	**<0.001** *
OSA	2577 (48.6%)	1198 (11.3%)	**<0.001** *
TBI	8 (0.2%)	54 (0.5%)	**<0.001** *
GLP-1 use	18 (0.3%)	33 (0.3%)	0.766*
Outcomes			
ADRD patients	6 female and 0 male: 6 (0.1%)	22 female and 7 male: 29 (0.3%)	**0.042** *
MCI patients	30 female and 17 male: 47 (0.9%)	81 female and 27 male: 108 (1.0%)	0.424*
Non-ADRD/MCI patients	5252 (99.0%)	10475 (98.7%)	0.126*
Death	65 (1.2%)	473 (4.5%)	**<0.001** *
Comorbidity after the baseline but before (1) the ADRD incidence (denominator: No. ADRD patients), (2) MCI incidence (No. of MCI patients), and (3) death/censoring (No. of non-MCI/ADRD patients)			
Hypertension			
Until ADRD	6 (100.0%)	25 (86.2%)	0.334*
Until MCI	39 (83.0%)	72 (66.7%)	**0.038** *
Until death/censoring	3095 (58.9%)	5082 (48.5%)	**<0.001** *
Dyslipidemia			
Until ADRD	2 (33.3%)	8 (27.6%)	0.777*
Until MCI	7 (14.9%)	18 (16.7%)	0.782*
Until death/censoring	322 (6.1%)	536 (5.1%)	**<0.001** *
IHD			
Until ADRD	3 (50.0%)	13 (44.8%)	0.817*
Until MCI	16 (34.0%)	22 (20.4%)	0.069*
Until death/censoring	372 (7.1%)	1104 (10.5%)	**<0.001** *
Depression			
Until ADRD	2 (33.3%)	9 (31.0%)	0.912*
Until MCI	24 (51.1%)	35 (32.4%)	**0.028** *
Until death/censoring	1104 (21.0%)	1417 (13.5%)	**<0.001** *
OSA			
Until ADRD	4 (66.7%)	13 (44.8%)	0.330*
Until MCI	26 (55.3%)	48 (44.4%)	0.213*
Until death/censoring	2416 (46.0%)	2376 (22.7%)	**<0.001** *
TBI			
Until ADRD	0 (0.0%)	3 (10.3%)	0.410*
Until MCI	2 (4.3%)	11 (10.2%)	0.221*
Until death/censoring	58 (1.1%)	142 (1.4%)	0.185*
GLP-1 use			
Until ADRD	0 (0.0%)	0 (0.0%)	—
Until MCI	0 (0.0%)	1 (0.9%)	0.508*
Until death/censoring	21 (0.4%)	105 (1.0%)	**<0.001** *
	Mean (SD)		
ADI at baseline	56.1 (18.7)	56.6 (19.2)	0.122$
CCI at baseline	0.53 (0.98)	0.50 (1.02)	**<0.001** $
Total weight loss, kg			
In 3 mo	17.2 (21.3)	0.24 (14.2)	**<0.001** $
In 6 mo	27.5 (22.7)	0.49 (12.9)	**<0.001** $
In 12 mo	36.5 (24.9)	1.24 (14.1)	**<0.001** $
In 3 y	33.5 (26.1)	1.47 (16.5)	**<0.001** $
In 5 y	23.9 (27.5)	2.13 (18.1)	**<0.001** $
In 10 y	20.8 (24.7)	2.24 (20.4)	**<0.001** $
Until ADRD diagnosis	30.9 (16.8)	6.42 (17.6)	**0.008** $
Until MCI diagnosis	18.8 (23.2)	3.14 (18.2)	**<0.001** $
Follow-up years			
Until ADRD	10.6 (5.4)	8.5 (5.3)	0.454$
Until MCI	6.7 (4.7)	5.4 (5.0)	**0.044** $
Until death	8.4 (5.2)	5.5 (5.0)	**<0.001** $
Until censoring	8.0 (5.5)	7.9 (5.5)	0.116$

* Proportion test.

† American Indian or Alaska Native, Asian, Hispanic or Latino, Native Hawaiian, or other Pacific Islander.

$ Mann-Whitney *U* test.

NA indicates Not Appropriate

Boldface shows that the differences in values between the surgery and control groups are statistically significant, as determined by the corresponding statistical tests.

The largest proportion of patients in both groups was observed in the BMI ≥ 50 kg/m^2^ category (31.98%), whereas the smallest proportion was in the 35 to 39.9 kg/m^2^ range (13.52%). The average age at baseline was 44.9 years, with an SD of 10.9 years for both cohorts, and most patients fell within the 18 to 45 age range (approximately 52.23%). The ADI average for both groups was around 56, where a ranking of 1 indicates the lowest and 100 the highest level of socioeconomic disadvantage nationally. Over 81.92% of participants were White, and the majority in both groups were female (over 80.48%).

Comorbidities within 1 year before baseline were more prevalent in the surgery group compared with the control group, specifically hypertension (65.58% vs 38.48%), dyslipidemia (9.82% vs 2.89%), depression (21.31% vs 7.64%), and OSA (48.60% vs 11.03%). We compared the same comorbidities after baseline between the surgery and control groups across three categories: comorbidities occurring after baseline but before ADRD incidence for ADRD patients; comorbidities after baseline but before MCI incidence for MCI patients; and comorbidities after baseline but before death or censoring for all non-ADRD/MCI patients.

We found that there were no significant differences in comorbidities after baseline but before ADRD incidence between the surgery and control groups among ADRD patients. However, among MCI patients, the surgery group had higher proportions of hypertension (82.98% vs 66.67%) and depression (51.06% vs 32.41%) compared with the control group. Among non-ADRD/MCI patients, the surgery group had higher rates of hypertension (58.93% vs 48.52%), dyslipidemia (6.13% vs 5.12%), depression (21.02% vs 13.53%), and OSA (46% vs 22.68%), and lower rates of IHD (7.08% vs 10.54%) and GLP-1 drug use (0.4% vs 1%) than the control group. The surgery group also exhibited much greater total body weight loss at 3, 6, and 12 months; 3, 5, and 10 years; and up to ADRD/MCI diagnosis compared with the control group.

ADRD incidence was observed to be higher in the control group (0.27%) compared with the surgery group (0.11%), with this difference being statistically significant (*P* = 0.04). Similarly, the incidence of MCI was greater in the control group (1.02%) than in the surgery group (0.89%), although this increase did not achieve statistical significance (*P* = 0.42). Furthermore, the control group experienced a higher mortality rate than the surgical group (4.45% vs 1.23%, *P* < 0.001).

In both the surgery and control groups, a higher number of females developed ADRD and MCI compared with males. Specifically, in the surgery group, all patients who developed ADRD were female (100% female vs 0% male). Among those who developed MCI in the surgery group, 63.83% were female and 36.17% were male. In the control group, among ADRD patients, 75.86% were female and 24.14% were male, while among MCI patients, 75% were female and 25% were male.

**FIGURE 1.**
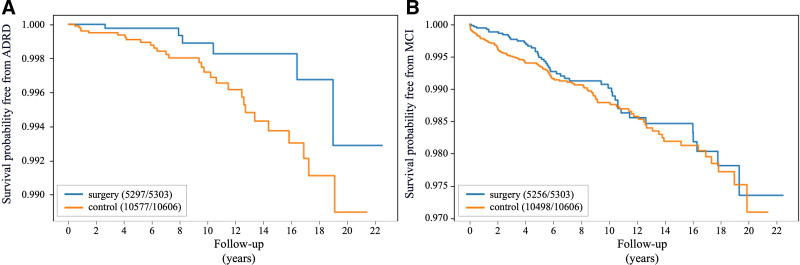
Kaplan-Meier survival curves for ADRD and MCI incidences. A, Survival probability free from ADRD, and (B) survival probability free from MCI, across the follow-up period for both the bariatric surgery and control groups.

Figure 1A and B highlights the probability of avoiding ADRD and MCI across the follow-up period for both the surgical and control groups. We used the variance inflation factor to detect the presence and severity of multicollinearity in our Fine-Gray models, setting a threshold of variance inflation factor <5 to address collinearity concerns. The log-rank test results showed a significant difference in ADRD incidence rates between the 2 groups, with an HR of 0.34 (95% CI: 0.12–0.92; *P* = 0.03) as presented in Table 2. Similarly, the Fine-Gray model indicated that bariatric surgery was associated with a reduced risk of ADRD, yielding an SHR of 0.37 (95% CI: 0.15–0.89; *P* = 0.02) (Table 2). Confounders such as hypertension within 1 year before baseline and IHD after baseline were associated with an increased risk of ADRD incidence. The incidence of ADRD was 0.11% in the surgical group and 0.27% in the control group. Linear regression results indicate the surgical group had 1.81 more follow-up years before developing ADRD compared with the control group (95% CI: −3.19 to 6.81; *P* = 0.46). The average follow-up period was 10.6 years for the surgical group and 8.5 years for the control group.

**TABLE 2.** HRs Using the Log-Rank Test and SHRs Using Fine-Gray Models

	SHR	*P*	HR	*P*
ADRD				
Bariatric surgery	0.37 (0.15–0.89)	0.028	0.34 (0.12–0.92)	0.034
Hypertension before the baseline	4.00 (1.54–10.40)	0.004	4.61 (1.65–12.90)	0.004
IHD after the baseline	2.76 (1.23–6.18)	0.013	2.78 (1.20–6.43)	0.017
MCI				
Bariatric surgery	0.57 (0.39–0.85)	0.006	0.57 (0.38–0.85)	0.007
CCI at baseline	1.21 (1.08–1.37)	0.002	1.21 (1.07–1.38)	0.003
ADI at baseline	0.99 (0.98–1.00)	0.004	0.99 (0.98–1.00)	0.005
OSA before the baseline	1.58 (1.02–2.46)	0.041	1.59 (1.01–2.52)	0.046
Depression after the baseline	1.89 (1.29–2.75)	0.001	1.90 (1.29–2.78)	0.001
TBI after the baseline	2.54 (1.39–4.64)	0.002	2.55 (1.44–4.53)	0.001

Bariatric surgery (RYGB or SG) was the primary factor of interest, and comorbidities showing statistical significance were also included.

The log-rank test results show a significant difference in MCI incidence rates between the 2 groups, with an HR of 0.57 (95% CI: 0.38–0.85; *P* = 0.01). Similarly, the Fine-Gray model indicates a SHR of 0.57 (95% CI: 0.39–0.85; *P* = 0.01), confirming the significant association. As shown in Table 2, patients undergoing bariatric surgery had higher CCI scores (0.53 vs 0.50), a higher prevalence of OSA within 1 year before baseline (48.60% vs 11.30%), and higher rates of depression after baseline but before MCI onset (51.06% vs 32.41%) compared with nonsurgery patients. These conditions are known risk factors for MCI and could potentially increase the overall MCI risk in the surgery group. The elevated MCI risk from these confounders may have masked the protective effect of bariatric surgery in the unadjusted analysis (Table 1). By adjusting for CCI, OSA, and depression, we were able to isolate the effect of bariatric surgery on MCI. This adjustment mitigated the influence of these confounding variables, allowing the protective association between bariatric surgery and reduced MCI risk to become apparent.

Linear regression revealed that the surgical group had 2.01 more years before developing MCI compared with the control group (95% CI: 0.70–3.50; *P* = 0.004). The incidence of MCI was 0.89% in the surgical group, with an average follow-up duration of 6.7 years, compared with 1.02% in the control group, which had an average follow-up of 5.4 years.

**FIGURE 2.**
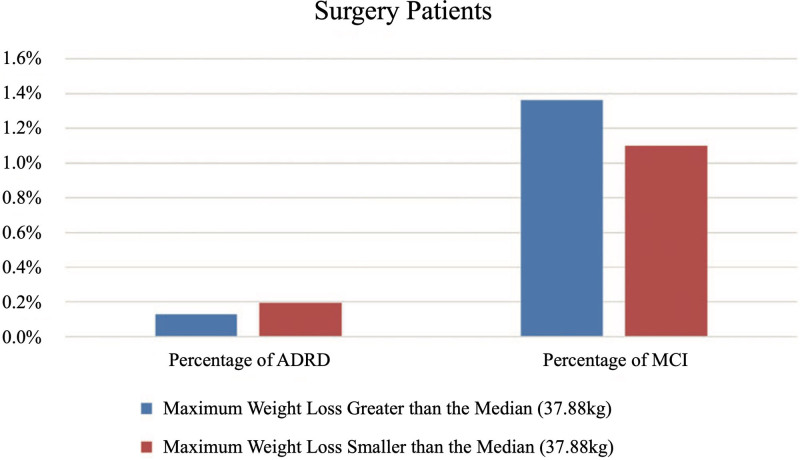
A comparison was conducted between the ADRD and MCI incidences in 2 patient groups: those whose maximum weight loss within 2 years postsurgery exceeded the median maximum weight loss of all surgery patients and those whose maximum weight loss was below the median. Statistical analysis revealed no significant differences in ADRD or MCI incidence between these groups.

Within the surgery group, we found that the maximum weight loss within 2 years after surgery was not associated with the risk of ADRD or MCI, as shown in Table 3. Patients who developed ADRD had a greater maximum weight loss (mean [SD]) of 42.3 kg (12.3 kg) compared with non-ADRD patients, who lost 37.6 kg (22.5 kg). Conversely, patients who developed MCI had a smaller maximum weight loss of 36.9 kg (26.3 kg) compared with non-MCI patients at 37.6 kg (22.5 kg). However, these differences were not statistically significant. To further investigate, we divided the surgery group into 2 subgroups based on the median maximum weight loss of 37.88 kg and compared the incidences of ADRD and MCI between them. The results, depicted in Figure 2, show no significant difference in ADRD incidence (greater vs smaller than median: 0.1% vs 0.2%) or MCI incidence (1.4% vs 1.1%). Chi-squared test results show there are no significant variations in ADRD (0.1% vs 0.1%) and MCI (1.0% vs 0.6%) risk between RYGB and SG surgeries.

**TABLE 3.** Statistical Analysis of the Association Between Maximum Weight Loss Within 2 Years Post Bariatric Surgery and the Incidence of MCI/ADRD in the Surgery Group

	Mean (SD), kg			
Maximum Weight Loss Within 2 y Postsurgery	ADRD	Non-ADRD	Coefficient	*P*
	42.3 (12.3)	37.6 (22.5)	0.0089	0.636
	MCI	Non-MCI		
	36.9 (26.3)	37.6 (22.5)	−0.0014	0.842

**FIGURE 3.**
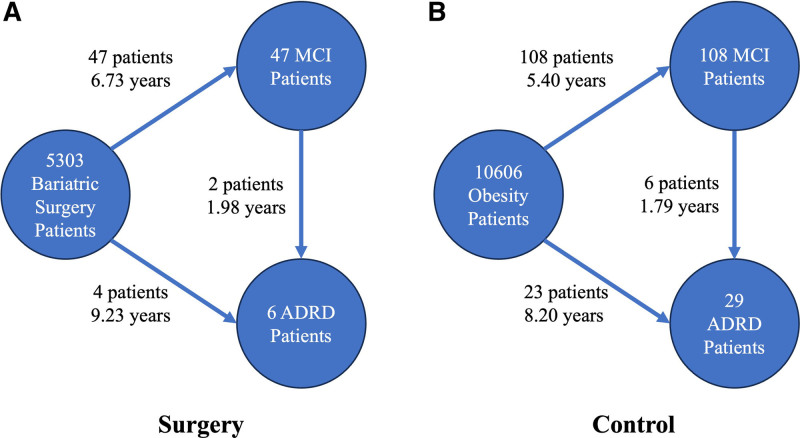
Summary statistics of the progression from MCI to ADRD in surgery and control groups.

**FIGURE 4.**
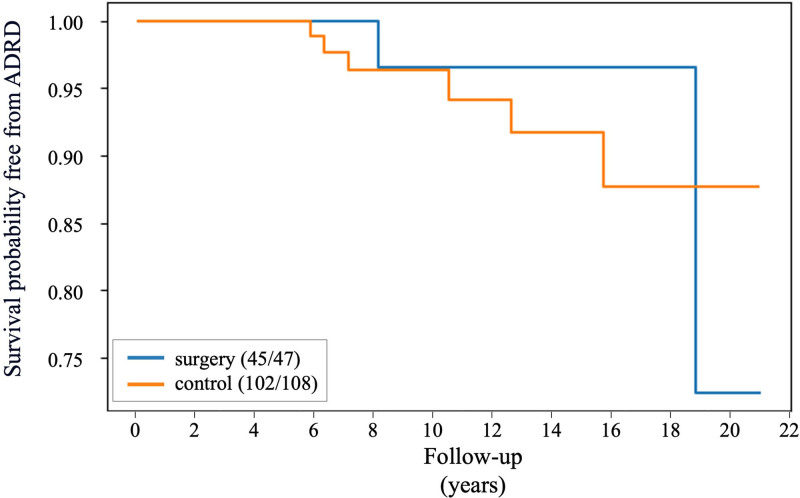
Kaplan-Meier survival curves for ADRD incidence among MCI patients. These curves illustrate the survival probability free from ADRD across the follow-up period for both the bariatric surgery and control groups.

We conducted an in-depth analysis of the incidences of ADRD and MCI to examine the progression from MCI to ADRD between the surgical and control groups. Figure 3A illustrates that in the surgery group, 2 of 47 patients with MCI (4.30%) advanced to ADRD over an average period of 1.98 years. Conversely, in the control group, 6 of 108 MCI patients (5.60%) progressed to ADRD over an average period of 1.79 years (Fig. 3B). Figure 4 shows the Kaplan-Meier curve for the survival analysis transitioning from MCI to ADRD in surgery and control groups. The differences in the transition probability from MCI to ADRD between the surgery and control groups did not reach statistical significance, with an HR of 0.73 (95% CI: 0.15–3.44) and a *P* value of 0.69.

## DISCUSSION

The use of decades of longitudinal EHR data provided a comprehensive observation window, enabling an examination of the long-term impact of bariatric surgery on cognitive health. The observed association between bariatric surgery and a lower risk of MCI and ADRD, in the context of increasing obesity rates and a rising global incidence of dementia, underscores the potential role of metabolic health improvements in cognitive health outcomes. It is important to clarify that our findings do not directly demonstrate that bariatric surgery leads to the prevention of MCI and ADRD. Instead, they suggest a correlation between bariatric surgery and a decreased risk of MCI and ADRD, which may reflect unmeasured variables such as differences in health behaviors, genetics, lifestyle, and environmental factors.

Bariatric surgery is associated with substantial weight loss, as evidenced by significant differences in total weight loss at 3, 6, and 12 months; at 3, 5, and 10 years; and up to the time of ADRD/MCI diagnosis between the surgery and nonsurgery groups (Table 1). While our findings suggest an association between bariatric surgery and a reduced risk of both ADRD and MCI, this effect cannot be conclusively attributed to weight loss alone. High rates of missing weight loss data postbaseline in both groups make it challenging to directly assess the impact of weight loss on ADRD/MCI risk. Within the surgery group, we found no association between the degree of weight loss (eg, weight loss above vs below the median) and the risk of ADRD/MCI, nor between the type of surgery—RYGB versus SG—and ADRD/MCI risk. This might indicate that the metabolic improvements following bariatric surgery, rather than the surgery type or degree of weight loss, play a more prominent role in reducing ADRD/MCI risk.

Improved metabolic health, particularly through better glucose control and reduced insulin resistance, might contribute to cognitive protection indirectly. Evidence suggests that hyperglycemia and insulin resistance are linked to neurodegeneration and cognitive decline,^23,39–41^ likely due to increased inflammation, oxidative stress, and vascular impairment in the brain. Therefore, it is plausible that bariatric surgery’s metabolic benefits might mitigate these cognitive risks, independent of weight loss magnitude. Similarly, weight loss surgery can modify hormone levels or profiles, such as ghrelin, leptin, and adiponectin,^24^ which are involved in hunger regulation, energy balance, and potentially brain health.^42,43^ These hormonal changes might offer neuroprotective benefits by affecting key processes such as neuroinflammation, neurogenesis, and brain metabolism. It is important to note that these are theoretical explanations based on the known functions of these hormones and their potential impact on the brain.^44,45^ While these hypotheses are grounded in biological plausibility, further research and references are needed to substantiate these potential mechanisms and to fully understand how bariatric surgery may influence cognitive health through hormonal alterations. Additionally, obesity is a well-known risk factor for cardiovascular diseases, which in turn are linked to increased dementia risk.^44^ By addressing obesity and associated conditions such as hypertension and dyslipidemia (the surgery group had a higher prevalence of these comorbidities compared with the control group: hypertension: 66.58% vs 38.50%, p<.001, and dyslipidemia: 9.82% vs 2.89%, *P* < 0.001), bariatric surgery diminishes vascular risk factors, potentially slowing down cognitive decline. Furthermore, weight loss management often encourages individuals to adopt healthier lifestyles, including increased physical activity and healthier dietary patterns, which have been independently associated with lower risks of cognitive decline.^45^ These lifestyle changes, facilitated by the surgery, might indirectly contribute to the observed cognitive benefits.

Bariatric surgery is associated with a reduced risk of ADRD, potentially through its influence on IHD. Although the prevalence of IHD within 1 year before baseline was similar between the surgery and control groups (5.07% vs 5.13%, not significant), we found that non-ADRD/MCI patients who underwent bariatric surgery had a significantly lower incidence of IHD after baseline but before death or censoring compared with controls (7.08% vs 10.54%). This suggests that bariatric surgery may confer cardiovascular benefits that reduce the incidence of IHD, a known risk factor for ADRD. Interestingly, among patients who developed ADRD, a higher percentage in the bariatric surgery group experienced IHD after baseline compared with the control group (50% vs 44.83%), though this difference was not statistically significant. Notably, of the 3 ADRD patients in the surgery group who developed IHD after surgery, 1 had a history of IHD before baseline. These findings highlight the complex interplay between metabolic health, cardiovascular disease, and cognitive decline. They suggest that while bariatric surgery may reduce the overall risk of ADRD partly by lowering the incidence of IHD, patients with pre-existing IHD or those who develop IHD after surgery remain at elevated risk for ADRD. Therefore, ongoing cardiovascular risk management is essential even after bariatric surgery to further mitigate the risk of cognitive impairment.

Our study reveals that bariatric surgery is associated with a reduced risk of MCI despite the surgery group exhibiting a higher prevalence of several confounders known to increase MCI risk. Specifically, patients who underwent bariatric surgery had significantly higher CCI scores at baseline (0.53 vs 0.50), indicating a greater burden of comorbid conditions. They also had a significantly higher incidence of OSA within 1 year before baseline (48.60% vs 11.30%) and higher rates of depression after baseline but before MCI onset among MCI patients (51.06% vs 32.41%) compared with the control group. Among non-ADRD/MCI patients, the surgery group continued to show higher rates of depression after baseline until death or censoring (21.02% vs 13.53%). Although TBI is a known risk factor for cognitive impairment and the surgery group had a lower percentage of TBI after baseline but before MCI onset compared with the control group, these differences were not statistically significant. Importantly, even after controlling for TBI in our analysis, bariatric surgery remained significantly associated with a reduced risk of MCI. These findings suggest that bariatric surgery may confer neuroprotective effects that reduce the risk of MCI, potentially outweighing the negative impact of higher comorbidity burden, OSA, and depression observed in the surgery group. The reduction in MCI risk might be attributed to the metabolic and inflammatory improvements following weight loss surgery, such as enhanced insulin sensitivity, reduced systemic inflammation, and improved vascular health, all of which are factors that can positively influence cognitive function.

The lower progression rate from MCI to ADRD observed in the surgical group (4.3%) compared with the control group (5.6%) suggests that bariatric surgery may slow the advancement of cognitive impairments to more severe forms of dementia; however, our test results did not reach statistical significance. An additional noteworthy aspect of our study is the sex differences in the incidence of ADRD and MCI in the surgery and control groups. Both groups had a higher proportion of female participants, with females constituting 80.48% and males 19.52% of the study population. In the surgery group, all patients who developed ADRD were female (100% female, 0% male), and among surgery patients who developed MCI, 63.83% were female and 36.17% were male. In the nonsurgery group, among ADRD patients, 75.86% were female and 24.14% were male, while among MCI patients, 75% were female and 25% were male. While these figures indicate a higher incidence of ADRD and MCI among females, it is important to consider that the overall study population was predominantly female. The proportion of females who developed ADRD and MCI is somewhat reflective of their representation in the study cohort. Despite the greater incidence of ADRD and MCI among females, the surgery group overall demonstrated a reduced risk of developing these conditions compared with the control group. This suggests that bariatric surgery may confer cognitive benefits that are especially impactful for female patients, potentially mitigating some of the sex-related risks.

However, this study is not without its limitations. First, the retrospective design, despite the comprehensive data available, cannot establish causality. Second, the reliance on ICD codes for diagnosing ADRD and MCI may either overestimate or underestimate the actual incidences. The accuracy of diagnosis based on ICD codes can vary, influenced by clinical practices, coding habits, and the sensitivity and specificity of the codes themselves. This methodological challenge highlights the need for a cautious interpretation of our findings and suggests an area for improvement in future research through the integration of more precise diagnostic tools and criteria. Additionally, while propensity score matching aimed to control for confounding variables, residual confounding cannot be entirely ruled out. Differences in follow-up duration and the inherent selection bias toward patients receiving bariatric surgery who might have been healthier or had better access to healthcare resources also pose challenges to generalizing these findings. Further, we did not account for the presence of micronutrient deficiency (eg, vitamins B and thiamin), substance use disorders, or autoimmune disorders that may be associated with the risk of ADRD or MCI due to limitations in the data and the complexity of the phenotyping processes. Finally, we did not investigate the association of bariatric surgery with each specific dementia subtype. Based on the data we have observed, we are unfortunately unable to achieve this level of detailed analysis.

Future studies should aim to overcome these limitations by incorporating prospective designs, more refined diagnostic criteria for cognitive impairments, and a broader set of covariates to better adjust for potential confounders. Longitudinal studies with more detailed cognitive assessments and metabolic monitoring postsurgery could further elucidate the mechanisms underlying the observed protective effects. Further investigation into potential mediators, such as inflammatory markers, hormone regulation, and neuroprotective mechanisms influenced by bariatric surgery, may provide deeper insights into the pathways linking obesity, weight loss, and cognitive health.

## ACKNOWLEDGMENTS

Y.C. and Y.F. had full access to all the data in the study and took responsibility for the integrity of the data and the accuracy of the data analysis. Concept and design; acquisition, analysis, or interpretation of data; critical revision of the manuscript for important intellectual content; and final approval of the version to be published: Y.C., Y.F., X.Z., K.A.G., Y.E., J.S., V.L.A., W.J.E., C.R.F., D.Y., R.Z., and S.I. Drafting of the manuscript and statistical analysis: Y.C. and Y.F. Obtained funding: Y.C. Supervision: S.I.
